# Evaluation of Soft Tissue Mobilization in Patients with Temporomandibular Disorder-Myofascial Pain with Referral

**DOI:** 10.3390/ijerph17249576

**Published:** 2020-12-21

**Authors:** Joanna Kuć, Krzysztof Dariusz Szarejko, Maria Gołębiewska

**Affiliations:** 1 Department of Prosthodontics, Medical University of Bialystok, 15-276 Bialystok, Poland; 2 Private Health Care, Physical Therapy and Rehabilitation, 15-201 Bialystok, Poland; biuro@rehabilitacja-lecznicza.pl; 3 Department of Dental Techniques, Medical University of Bialystok, 15-269 Bialystok, Poland; maria.golebiewska@umb.edu.pl

**Keywords:** electromyography, masticatory muscles, myofascial pain with referral, soft tissue mobilization, orofacial rehabilitation, temporomandibular joint

## Abstract

The aim of the study was functional evaluation of soft tissue mobilization in patients with temporomandibular disorder-myofascial pain with referral. The study group consisted of 50 individuals—37 females and 13 males. The average age was 23.36 ± 2.14 years. All subjects were diagnosed with myofascial pain with referral (diagnostic criteria for temporomandibular disorders). Soft tissue mobilization was applied three times. Electromyography of selected masticatory muscles was performed six times—before and after the treatment. After each mobilization, a decreasing tendency of muscular activity was observed in the entire study group. The Friedman test indicated that mobilization altered the activity of the right temporal muscle (*p* = 0.00010), both masseters (*p* = 0.0000), right sternocleidomastoid (*p* = 0.00251), left sternocleidomastoid (*p* = 0.00033), and right and left digastric muscles (*p* = 0.00045 and *p* = 0.00000, respectively). With respect to symmetry a statistically significant difference was noted in the case of the sternocleidomastoid muscles (*p* = 0.00729). In conclusion, soft tissue mobilization seems to be effective in the relaxation of masticatory muscles in patients with temporomandibular disorders. Our findings proved that soft tissue mobilization does not improve the symmetry and synergy of the masticatory muscles limited by dental occlusion.

## 1. Introduction

Myofascial pain was first described in 1952 by Travell and Rinzler [1]. This pain is a psychophysiological disorder associated with muscle tissue, particularly the muscles of the masticatory system [2]. In the classic approach, myofascial pain is conditioned by the presence of trigger points (TrPs) [2,3,4]. They appear locally or at a certain distance from the place initially affected by the disorder [2]. This aspect differentiates myofascial pain from other kinds of pain in the musculoskeletal system [2].

According to Jafri, myofascial pain is a combination of sensory experiences, motor reactions and autonomic symptoms, including local and projected pain. Its typical manifestations include a limited range of movement, impaired coordination and muscle weakness [5]. Mood disorders and decreased quality of life are observed in addition to the risk of functional deterioration [5,6].

The cause of myofascial pain may include micro and macrotraumas, overloads leading to chronic muscle fatigue, improper posture, poor work ergonomics, degenerative changes accompanying the process of ageing and the resulting loss of myofascial elasticity and nerve root compression that enhances sensitization of spinal cord structures [2]. Attention is drawn to the use of calcium channel blockers and systemic factors, i.e., increased emotional tension, endocrine problems, sleep disorders, nutritional deficiencies and infections [7]. An important role is attributed to pathological occlusion and abnormal prosthetic restorations [2]. Moreover, researchers quote the parafunctional use of the mouth, jaw or tongue, perioral habits, tooth fractures and craniomandibular dysfunction associated with skeletal disorders [7]. Myofascial pain may also be a postoperative consequence of tooth extraction [2]. This type of pain is conditioned by stress factors, and appears to be the most common cause of pain symptoms in temporomandibular joint disorders [3,8]. It is also one of the main causes of chronic symptoms in the craniofacial region [3,9].

Myofascial pain probably results from hypothyroidism, parasitic diseases, babesiosis, Lyme disease, recurrent fungal infections, deficiency of vitamins D and B12, low levels of iron and hormonal disorders in both sexes [4,10,11].

The main causes of myofascial pain are: chronic emotional stress, impaired functioning of the descending inhibitory system, sleep deprivation, specific unhealthy behavior and depression [12]. These factors are of a medical nature and tend to strengthen the main disease. Separate categories of myofascial pain causes include structural determinants, i.e., scoliosis, physique abnormalities, limb length disorders, torsion or pelvic asymmetry, sacroiliac and hip joint dysfunctions and spine degenerations [4]. Another category are ergonomic factors associated with hypermobility, head and shoulder protraction, pathological activity resulting from work, long-term static positions, a highly energetic pattern of constantly repeated activities, activity associated with stress and working in front of a computer and disturbances with significant movement limitation, i.e., Duplay’s disease [4,13].

In numerous cases the elimination of the above-mentioned factors may lead to the deactivation of trigger points and reduction of the pain. Their early diagnosis and subsequent exclusion determine therapy success in patients with chronic pain [7].

Considering that soft tissue mobilization is associated with changes of myofascial balance it was hypothesized that there is at least one significant difference between pre- and post-treatment activity of selected muscles of the stomatognathic system in patients with temporomandibular joint disorder-myofascial pain with referral.

The aim of our study was functional evaluation of soft tissue mobilization in patients with temporomandibular joint disorder-myofascial pain with referral.

## 2. Materials and Methods

### 2.1. Ethical Issues

The study was approved by the Ethics Committee of the Medical University of Bialystok, Poland (permission number: R-I-002/322/2016). The research was conducted in accordance with the principles of the Declaration of Helsinki of the World Medical Association and the Guidelines for Good Clinical Practice. Participation in the study was voluntary. All the patients obtained comprehensive information about the nature, scope of clinical activities and the course of the proceedings. At each stage, the subjects had the right to refuse to participate in the research without any resulting consequences. Participation in the study had been preceded by a written consent by every patient.

### 2.2. Subjects and the Size of the Sample

The research was conducted in the Department of Prosthodontics at the Medical University of Bialystok, Poland, on a group of 50 generally healthy individuals. Participating in the study were 37 females and 13 males. The average age of the subjects was 23.36 ± 2.14 years. The range of age was 18–25 years. All participants underwent a clinical examination with respect to the diagnostic criteria for temporomandibular disorders and were diagnosed as patients with myofascial pain with referral (axes I and II) [14].

#### 2.2.1. Inclusion Criteria

- Pain in the craniofacial and/or craniomandibular areas at the level of 8 points or more with respect to VAS (visual analog scale);
- Full natural dentition (class I according to angle’s molar classification and canine relations);
- Lack of orthodontic treatment history or retention status after completion of treatment exceeding 3 years.

#### 2.2.2. Exclusion Criteria

- Traumas or surgical procedures within the craniofacial region;
- Occlusal splint therapy;
- Prosthetic treatment before recruitment to the study;
- Metabolic diseases;
- Cases in which medication or possible health concerns could affect the functioning of the masticatory muscles;
- Previous physiotherapeutic treatment within the craniofacial, craniomandibular and/or craniocervical areas.

The presence or absence of third molars was not an inclusion/exclusion criterion in our study. The sample has been previously described in another paper [15].

### 2.3. General Description of the Method

All subjects underwent a thorough examination. The procedure included:

- Functional examination of temporomandibular joints and muscles of the stomatognathic system with respect to the diagnostic criteria for temporomandibular disorders (DC/TMD) [14]—axes I and II;
- Electromyography (BioEMG, BioResearch, Inc., Milwaukee, WI, USA);
- Soft tissues mobilization;
- Statistical analysis using the Statistica 13.1 software (TIBCO Software Inc., Statsoft, Cracow, Poland), IBM SPSS Statistics 26.0 (IBM Corporation, Warsaw, Poland) and PQStat Software v. 1.6.8. (PQStat Software, Poznan, Poland)

### 2.4. Electromyography (EMG)

**Figure 1:**
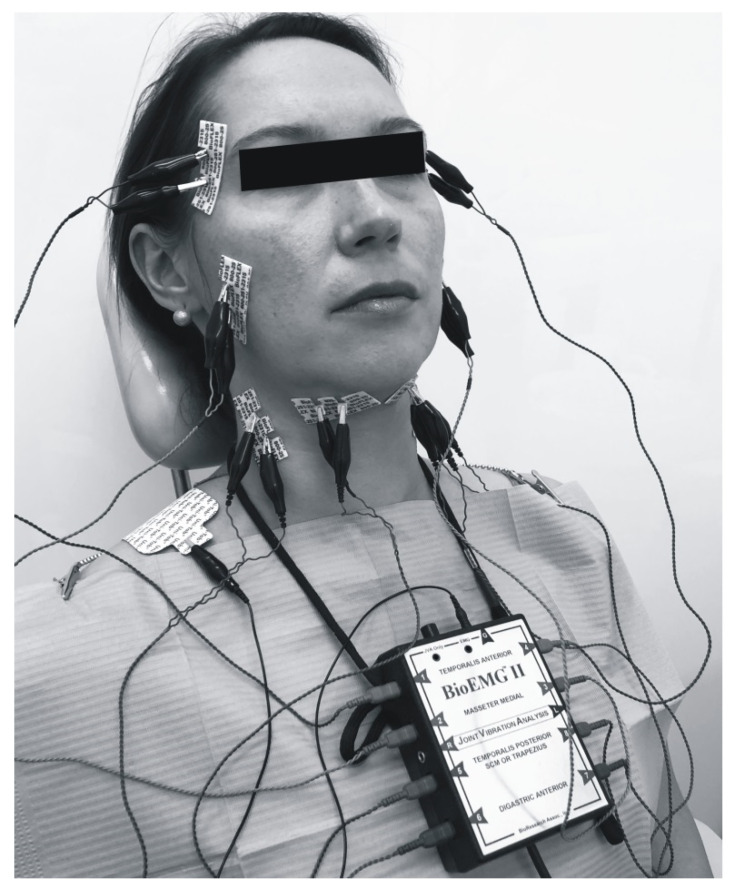
BioEMG (eight channel device for recording the muscular activity) and electrode locations.

An electromyographic examination was performed 6 times, always before and after the 1st, 2nd and 3rd soft tissue mobilization. Activity of the anterior part of the temporal muscles, masseter, sternocleidomastoid muscles and venter anterior of digastric muscle was assessed (Figure 1). Three registrations of individual potentials were performed at maximum intercuspation. During a single EMG recording, the patients were asked to clench both dental arches together, maintain this position for about 2 s, and then open their mouth wide and repeat these steps 3 times at 1-s intervals. There were 1-min breaks between the subsequent registrations. The activity of the selected muscles, symmetry and synergy in their respective functional groups were evaluated.

In the clinical procedure, Ag/AgCL bipolar gel electrodes with a constant interpolar distance (19 mm) were used (BioFLEX EMG Electrode, Bioresearch Inc. Milwaukee, size 4.5 cm × 2 cm). Electrode locations were determined by palpation over the muscle bulk during contraction, maintaining parallelism with the course of muscle fibers. A parallel position was maintained between the electrodes used to examine the temporal and masseter muscles. In the case of electrodes testing the activity of the anterior temporal muscles, the rule of 2-finger width (index and ring fingers) was applied for a distance from the outer edge of the eyebrow. For the masseter muscles the rule of the right angle between the thumb and index finger was implemented. In this case, the thumb was directed towards the electrode within the masseter muscle, and the index finger was in line with the mandibular plane. The right hand was used for the right facial profile, and the left hand—for the left side.

The reference electrode was located in the right supraclavicular fossa. In order to reduce impedance, the facial skin was rinsed with 2% salicylic alcohol. Electromyography was performed in the morning, under the conditions of suggestive relaxation (no background noise, no side conversations, no third parties accompanying the patient, a lack of visual (e.g., monitor light), auditory (e.g., the radio) and multisensory (audio-visual; e.g., smartphones) distractors). The entire procedure was performed by an experienced dentist specialized in prosthodontics and a physiotherapist in one person (the author J.K.). During the registration, the patients remained upright, sitting in the initial default position of the dental chair.

### 2.5. Soft Tissue Mobilization

**Figure 2:**
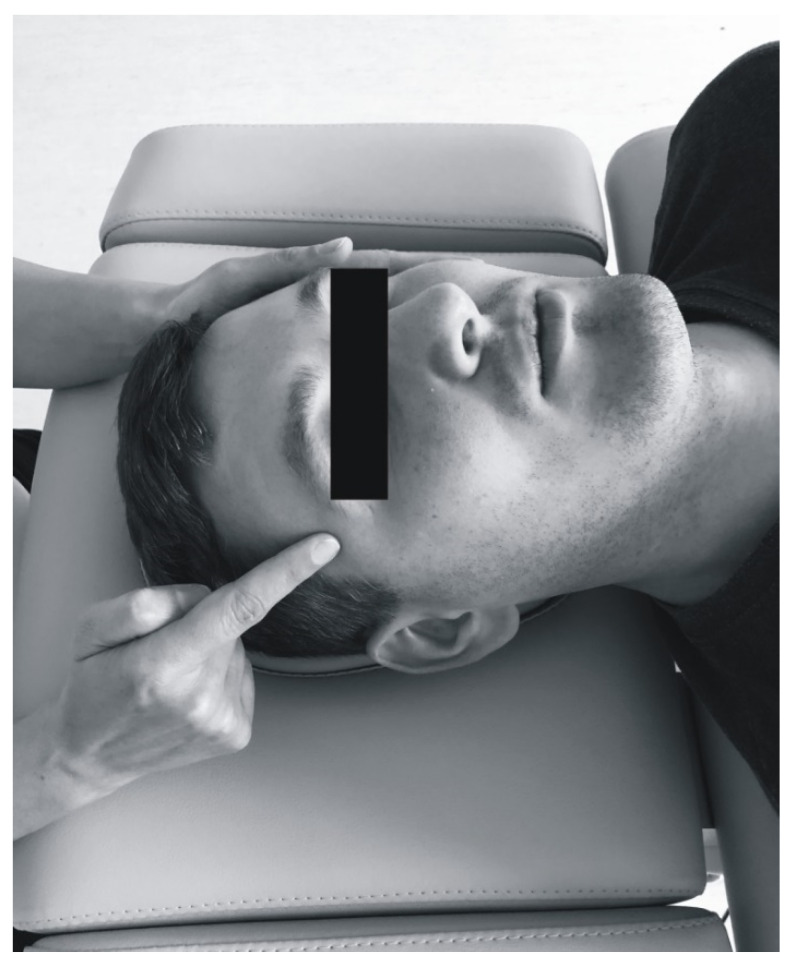
Unilateral soft tissue mobilization of the temporal muscle.

**Figure 3:**
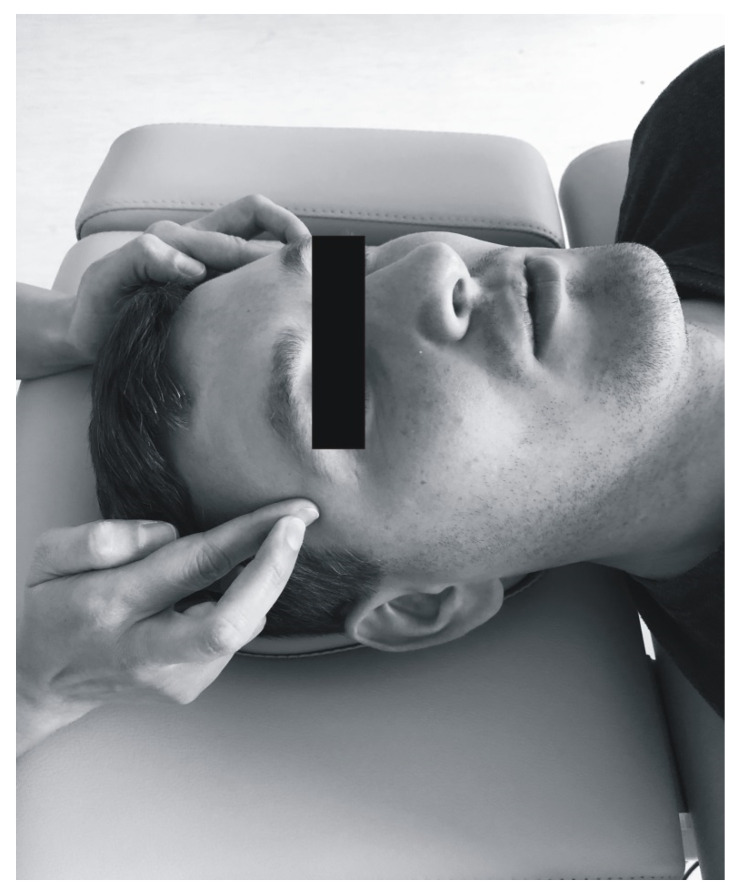
Bilateral soft tissue mobilization of the temporal muscles.

**Figure 4:**
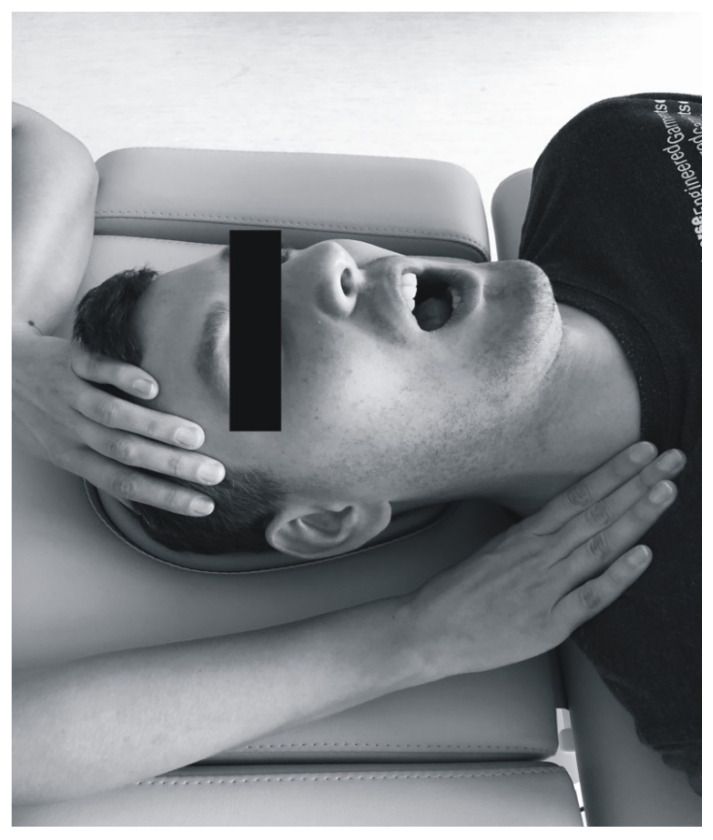
Soft tissue mobilization (upward—downward technique for masseter and temporal muscles; combined technique) [3].

**Figure 5:**
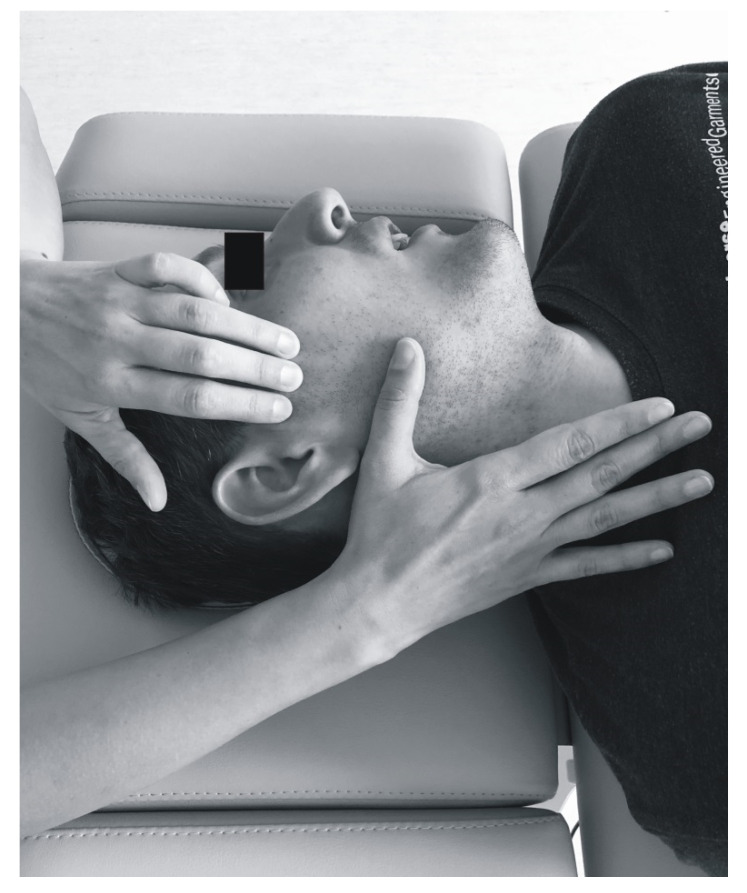
Soft tissue mobilization (masseter-oriented technique) [3].

All the patients underwent soft tissue mobilization for 30 min (Figure 2, Figure 3, Figure 4 and Figure 5). The procedure was performed three times at weekly intervals, under constant test conditions. The treatment of trigger points within the masseter and temporal muscles, and the myofascial relaxation technique, were applied. The clinical procedures were conducted by an experienced physiotherapist specialized in general physiotherapy (the author K.D.S.). The patient laid down on the table for manual therapy and the therapist remained above the head of the treated person. The physiotherapist released the patient in general silence, in a quiet room with no potential distractions.

**Figure 6:**
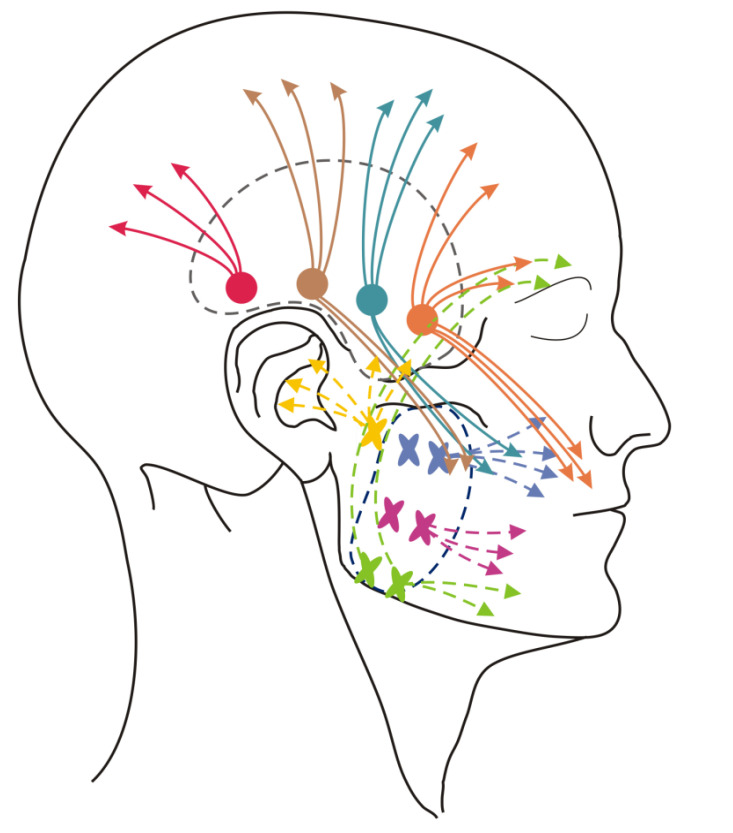
Trigger points of the masseter (marked by yellow, green, purple and cyan × signs) and temporal muscles (marked by pink, dark brown, blue and light brown dots) and patterns of referred pain (marked by arrows) according to Travel and Simons [3].

Trigger point pressure release was performed with respect to the trigger points within the masseter and temporal muscles (Figure 6) [3]. The pincer method was implemented to detect trigger points in the superficial layer of the masseter muscles. One finger remained inside the mouth within the cheek, while the other was outside. Muscle palpation was performed perpendicularly to the direction of the fibers in order to detect taut bands. External flat palpation was used for the deep layer of the masseter muscle. The same technique was applied to temporal muscles.

The therapy of trigger points involved initiating a slow pressure increase over the active trigger point until the tissue barrier was reached [3]. One finger for contacting the trigger point and one hand for contralateral stabilization of the head were used (Figure 2). Regarding bilateral active trigger points, both hands provided simultaneous treatment (Figure 3). Soft tissue mobilization started at the anterior margin of the muscle and moved posteriorly. The treatment was focused on the muscle areas that required release. The physiotherapist lengthened the muscle to the extent allowed by the patient’s comfort. Gentle, gradually increasing pressure on the trigger point was applied until the finger reached a marked increase in tissue resistance (involved the barrier). It was acceptable for the patient to experience some discomfort, but not pain [3]. Such pressure was maintained (but not increased) until the physiotherapist felt the tissue released under the touching finger. Then the clinician increased the pressure again. The slack of the soft tissue was appropriately selected to achieve a new barrier (the finger followed the releasing tissue). Next only light pressure was applied again to obtain more muscle tension release under the finger. At that stage, to achieve a better effect it was considered acceptable to change the direction of pressure [3]. The trigger point pressure release technique is tailored to the needs of the individual patient’s muscles and can be repeated for each band of the taut muscle [3]. The procedure was repeated until tenderness and/or tension of the muscles was reduced, or for 1.5 min, depending on what occurred first.

Myofascial relaxation was used to functionally release fascia. The physiotherapist placed one hand over the temporal muscle and took up the slack by applying upward traction (Figure 4) [3]. The other hand completed myofascial release by slow downward traction. Soft tissue release proceeded from the temporal muscle through the masseter towards the platysma. In the second masseter-oriented technique, the physiotherapist stabilized the muscle attachment in the zygomatic arch with one hand (Figure 5) [3]. The other hand moved along the muscle fibers, from the zygomatic arch to the edge of the mandible. Slight pressure was simultaneously applied to the back of the mandible to select slack within the masseter. During this procedure, the patient was asked to open his/her mouth wide and breathe deeply in order to augment muscle release [3]. After 1.5–2 min of interaction, initial balance was obtained. Then the therapist proceeded to another point of tissue resistance. The procedure was repeated several times (about 2–5 cycles) until the tension was reduced.

### 2.6. Statistical Analysis

Statistical analysis was performed using the Statistica 13.1 software (TIBCO Software Inc., StatSoft, Cracow, Poland), IBM SPSS Statistics 26.0 (IBM Corporation, Warsaw, Poland) and PQStat Software v.1.6.8 (PQStat Software, Poznan, Poland). All the studied parameters (activity of selected muscles of the stomatognathic system, muscular synergy and symmetry) were assessed in 6 periods—directly before and immediately after all three soft tissue mobilizations.

Central tendency measures, including arithmetic mean and median and measures of differentiation, i.e., standard deviation, were presented. The Friedman test was used for repeated measures to determine whether soft tissue mobilization has an effect on the activity, symmetry and synergy of selected muscles of the stomatognathic system. Friedman statistic (F_r_), degrees of freedom (df), *p*-value and Kendall’s coefficient of concordance (W) were presented. The results for the test probability level of *p* < 0.05 were considered statistically significant. Kendall’s coefficient of concordance (W) at the level of 0.1 indicated a small effect size, 0.3—moderate effect and results above 0.5—strong effect size. A Dunn–Bonferroni post hoc test was used to subsequently compare the different treatments following the Friedman test.

## 3. Results

Soft tissue mobilization reduced the activity of selected muscles of the stomatognathic system in the entire study group (*n* = 50; Table 1). Before the treatment, the average activity of the right temporal muscle was 108 µV, and of the left one, 93.1 µV. After the first mobilization, bioelectrical activity decreased to 82.3 µV on the right and to 80.5 µV on the left side. For the right masseter muscle, the initial value was 182.7 µV and for the left muscle, 168.7 µV. After the first treatment, the activity of the right masseter was 128.9 µV and of the left one, 129.9 µV. A decreasing tendency was also observed in the sternocleidomastoid and digastric muscles (Table 1). For the entire study group, the Friedman test indicated that soft tissue mobilization altered the activity of the right temporal muscle, both masseters, both sternocleidomastoids and both digastric muscles (*p* < 0.05; Table 1). For most muscles, the value of Kendall’s W coefficient was at least 0.1 points. Only for the right masseter muscle this parameter amounted to 0.3. A Dunn–Bonferroni test revealed that in the activities of the right temporal muscle, both masseters, both sternocleidomastoids and both digastric muscles there were statistically significant differences before the first and second soft tissue mobilization and the first and third treatment (*p* < 0.05 adjusted to the Bonferroni correction; Table 1). Among pre- and post-treatment results of the first therapy there were no statistically significant differences in the activities of both sternocleidomastoid muscles and the right digastric muscle (*p* > 0.05 adjusted to the Bonferroni correction; Table 1).

In the group of females, a decreasing tendency of muscle activity was noted (Table 2). The Friedman test revealed statistically significant differences among the mean ranks of activity of the right temporal muscle, both masseters, both sternocleidomastoid and both digastric muscles (*p* < 0.05; Table 2). A Dunn–Bonferroni test revealed that in the activities of the right temporal muscle, both masseters, both sternocleidomastoid and both digastric muscles there were statistically significant differences before the first and third soft tissue mobilizations (*p* < 0.05 adjusted to the Bonferroni correction; Table 2). In the first treatment, there were no statistically significant differences in pre- and post-treatment activities of the right temporal muscle, left sternocleidomastoid and right digastric muscle (Table 2). The null hypothesis could not be rejected in case of the left temporal muscle (Table 2). In the activities of the right sternocleidomastoid and right digastric muscles, there were no statistically significant differences noted before the first and after the second soft tissue mobilization (Table 2).

In the group of males, the Friedman test revealed statistically significant differences among the mean ranks of activity of both masseters and the left digastric muscle (*p* < 0.05; Table 3). Statistically considerable differences in the activity of these muscles before the first and second soft tissue mobilizations were observed (*p* < 0.05 adjusted to the Bonferroni correction; Table 3). Additionally, the right masseter muscle activity before the first and after the third treatment also differed considerably (Table 3).

In the entire study group, the symmetry in activity of the temporal muscles was 81.9% before the first soft tissue mobilization (Table 4). For the masseters this parameter remained at the level of 80.1%. Before the first mobilization, the symmetry in the activity of the sternocleidomastoid muscles was 72.0%, and for the digastric muscles: 82.4%. The synergy of the activity of the temporal and masseter muscles on the right and left side was similar and amounted to 60%. The Friedman test revealed statistically significant differences only among the mean ranks of symmetry in the activity of the sternocleidomastoid muscles (*p* < 0.05; Table 4). According to the Dunn–Bonferroni post hoc test, there was a statistically significant difference only between the symmetry in the activity of the sternocleidomastoid muscles registered before the first and after the third soft tissue mobilization (*p* < 0.05 adjusted to the Bonferroni correction; Table 4). A similar trend was observed in the group of females (Table 5).

In males, the Friedman test indicated statistically significant differences connected with the symmetry in the activity of the temporal and sternocleidomastoid muscles (Table 6). A Dunn–Bonferroni test revealed statistically significant differences of the symmetry in the activity of the temporal muscles before and after the third soft tissue mobilization (*p* < 0.05 adjusted to the Bonferroni correction; Table 6).

## 4. Discussion

Currently, treatment of patients with temporomandibular joint disorder is an interdisciplinary challenge. Clinical procedures used for years are increasingly expanded with new, often non-invasive, reversible methods. They usually include soft tissue techniques, mobilization of the temporomandibular joint, active or passive stretching, isometric tension exercises against resistance or guided opening and closing jaw movements [16]. The most commonly used treatments for the myofascial pain with referral involve myofascial release, the muscle energy technique, compression, acupressure and soft tissue mobilization [17,18].

The main goal of the manual therapy is to ease the tension of the masticatory muscle and joint pain, and to improve muscle coordination [16]. Shafer et al. highlighted that soft tissue mobilization should not be overlooked in the treatment of temporomandibular disorders [19]. These authors indicated that soft tissue mobilization should be applied to the temporal and masseter and both medial and lateral pterygoid muscles [19]. Sometimes, additional masticatory muscles and cervical spine could also be considered [19]. Bialosky et al. suggested that manual therapy is associated with neurophysiological responses from the peripheral and central nervous system, which indirectly implicates specific clinical outcomes [20]. This neurophysiological mechanism can influence pain modulation and leads to muscular activity reduction [21,22,23,24].

Carlsson et al. demonstrated that stretch-based relaxation enabled greater reduction of the activity of both masseter muscles than postural relaxation [25]. Biasotto-Gonzalez et al. noted that massage techniques decreased the activity of the temporal and masseter muscles to a statistically significant extent [26]. Capellini et al. highlighted that massage pressure had an inhibiting effect on the motor neurons, but this effect wears off quickly [27]. Following others, these authors, again emphasized that reflex responses excited in the periphery by palpation have only moderate influence on the muscular activity mediated by the healthy central nervous system [27]. Ginszt et al. showed that the compression technique applied to masseter muscles with active trigger points decreased their resting bioelectrical activity. During maximum voluntary contraction the opposite effect—an increased tendency—was observed [18]. Manzotti et al. demonstrated that osteopathic manipulative treatment (OMT) results in improved activity of the temporal and masseter muscles in 40% of the subjects [28]. These authors recommend OMT as a valid aid in preparation for a gnathological intervention. An appropriate balance of muscular tension and release of postural compensation could be achieved [28].

These study results showed that, statistically significant differences observed with respect to the first, second and third soft tissue mobilization may indicate the powerful impact of the central nervous system (CNS), and thus the muscle engrams conditioning a specific pattern of muscular activation (Table 1). According to the latest research, the time needed to extinguish engrams, contrary to the previously held belief, can only be two minutes [29]. This may in turn explain the effect of immediate changes in the activity observed after the first treatment of the right temporal, both masseter and left digastric muscles and in all the examined muscles after the second and third soft tissue mobilization, except the left temporal muscle. For the initially registered muscular activities, the third soft tissue mobilization proved the most effective (*p* < 0.001; Table 1). It should be emphasized that each mandibular motion to the position of the maximal intercuspation strengthens the muscle engrams, the so-called muscle memory, which may affect the typical activation of the anchored muscle formula [29]. This situation could explain the fleeting nature of the re-educational strength-forced neuromuscular facilitation of the masticatory muscles limited by the dental occlusion.

In females, statistically significant differences in the muscle activity may indicate the main source of dysfunction, pointing to the subtle neuromuscular discoordination. The general direction of changes was similar to that observed in the entire study group (Table 2). Among the males, statistically significant differences in the activity of the right masseter muscle were recorded after the second and third soft tissue mobilization (Table 3). The function of the left masseter muscle was considerably affected by the second treatment (Table 3). In the case of both temporal and sternocleidomastoid muscles, and the right digastric muscle soft tissue mobilization did not entail statistically significant differences (Table 3). The direction of changes in the group of males was varied. It should be emphasized that mean values of muscle activities registered in the entire study group, both in the group of females and males, were considerably lower than those observed by other authors (Table 1, Table 2 and Table 3) [30]. Decreased muscle activity could be a consequence of pain, which may lead to changes in muscle functions and limit mandibular movements [18]. Alajbeg et al. noted that in young people (26.7 ± 2.8 years) with natural dentition the average activity of the right and left masseter muscles amounted to 198.1 µV and 228.5 µV respectively. In turn, the activity of the right and left temporal muscles was at the level 239.2 µV on the right and 239.8 µV on the left side [30].

In the entire study group, high values of symmetry of muscular activity (>80%) were observed in the case of the temporal and masseter muscles, both before and after the treatment cycle (Table 4). The results for digastric muscles oscillated at a similar level (Table 4). Slightly lower values—although satisfactory—were obtained within sternocleidomastoid muscles (>70%; Table 4). Slightly lower values—but still satisfactory—were obtained within sternocleidomastoid muscles (>70%; Table 4). The synergy of the temporal and masseter muscles of both sides (right and left) was at a mid-range level (reference value 50–70%). In reference to soft tissue mobilization, dental occlusion seems to be the limitation factor preventing optimal symmetry and synergy as a desirable manual therapy results. This suggestion appears in line with the above-mentioned recommendations of gnathological intervention proposed by Manzotti et al. [28]. This aspect is also related to the engram theory.

Ries et al. observed that in patients with temporomandibular disorders the symmetry of the temporal muscle at maximum intercuspation was 95.03% ± 0.58% [31]. The average value for the masseters was 94.87% ± 0.43%, and for sternocleidomastoid muscles: 78.03% ± 1.24%. Ries et al. demonstrated that in subjects with temporomandibular disorders the symmetry in the activity of the masseter and sternocleidomastoid muscles was lower than in the control group (*p* < 0.05) [31]. These authors indicated that asymmetry during the activity of the masticatory muscles (e.g., excessive tension on the side of laterotrusion and maintenance of head extension) leads to faster fatigue and contributes to pain. The consequence is the activation of compensatory motor reactions, which can alter the habitual position of the mandible, the individual masticatory pattern, or the cervical spine setting [31]. These authors also noted that reinforced and improved sensory discrimination in the craniofacial region is determined by the contraction of the masticatory muscles, which positively affects proprioception during mandibular movements. The ability to properly distinguish the arriving stimuli enables the control of muscle condition [31]. Therefore, it is important to maintain balanced neuromuscular activity, including optimal symmetry of the temporal and masseter muscles at maximum intercuspation [31]. These observations coincide with Lerman’s conclusions on muscle engrams [29].

Ries et al. observed the highest asymmetry of the temporal and masseter muscles in the resting position of the mandible, when the feeling of proprioception is weakened due to the lack of dental contacts [31], and low values of symmetry and synergy could be expected. According to these authors, the position of maximum intercuspation and the individual repetition of the chewing cycle improve the proprioception and muscle stability [31].

Tartaglia et al. showed that in the group of patients with temporomandibular joint disorders the most appropriate symmetry of the temporal and masseter muscles (84.9% ± 5.8% and 83.6% ± 7.6%, respectively) was noted in individuals with a myogenic component [32]. In the group with degenerative changes of the temporomandibular joint, the symmetry of the temporal and masseter muscle amounted to 82.7% ± 7.1% and 83.3% ± 8%, respectively. In patients with a psychogenic component, the symmetry of the temporal muscle was 80.5% ± 12% and the masseter: 81.7% ± 9.3% [32].

It should be emphasized that the dysfunction of temporomandibular joints is the second cause of pain in the craniofacial area after tooth derangement [33]. Only 3% of patients who are aware of the symptoms seek medical help [33]. According to the latest reports, there is a close relationship between temporomandibular joint disorders and dysfunction of the cervical spine [33]. Nonetheless, Von Piekartz et al. demonstrated that six sessions of musculoskeletal approaches performed within 4–6 weeks (accessory movements, tender and trigger points and stretching of muscles) were more effective than a manual therapy applied within the cervical spine [34]. An important role is attributed to the mechanisms of central sensitization of pain and to biopsychosocial aspects [33]. Despite numerous studies, the available evidence-based medicine for soft tissue mobilization is still very limited. It is well known that soft tissue mobilization is crucial in the treatment of TMD patients due to its importance for all TMD diagnostic classifications [19]. Cuccia et al. noted that myofascial release, muscle energy, thrust and craniosacral therapy were more effective than oral appliance [35]. Similar tendency was observed by Ismail et al. with respect to splint therapy 24 h a day [36]. Lopez et al. showed that combined therapy, i.e., manual techniques and splint, resulted in more effective pain reduction, higher pain pressure threshold, improvement of disability and better perception, compared to the splint therapy alone [37]. Calixtre et al. observed increased effectiveness of myofascial release and massage within the masticatory muscles. The techniques for manipulating or mobilizing the upper cervical spine seem to have a similar effect. In turn, thoracic manipulations do not offer such results [38]. According to other studies, also cannabidiol (CBD) formulation reduces the activity of the masseter muscles and improves the muscular condition in patients with myofascial pain [39].

In the presented study, soft tissues mobilization probably contributed to the reduction of sympathetic nervous system activation and lowering of muscle hypertonia, thus leading to changes in the EMG registration and reduction of pain [40]. Differences in bioelectrical activity are the result of transient neuromuscular release (myofascial relaxation). The simultaneous release of metabolites and improvement of blood flow reduce the perceived discomfort. This is favored by the general state of relaxation of the patient conditioned by the applied therapy.

### Strengths and Limitations of the Study

The DC/TMD protocol enables the qualification of patients for the study in relation to strictly defined research criteria. It allows for better communication between scientists conducting research in similar fields and also enables comparison of the results of similar studies regarding their subject. The clear DC/TMD protocol and the clinical standards of BioEMG registration ensure research repeatability. As an extension of the DC/TMD concept, this study additionally included instrumental analysis, clinical intervention (soft tissue mobilization) and reassessment. The observed research findings could be referred to patients with temporomandibular disorders, including myofascial pain with referral. The main goal of our clinical intervention was to help patients reduce their pain, and this goal was achieved.

However, it should be borne in mind that DC/TMD protocol and instrumental analysis are time-consuming. Moreover, answers of self-reported questionnaires (DC/TMD) could be exaggerated. Sometimes patients are too embarrassed to reveal their emotions, social relations and other details. DC/TMD protocol contains an abundance of information, which makes it difficult to present all data in one publication. Extending the protocol by the inclusion of instrumental analysis and clinical intervention requires the generation of even more data. The aim of using the DC/TMD protocol in scientific research is usually to establish a diagnosis that could provide a more homogeneous study group.

The advantage of BioEMG is the fact that surface electromyography is a widely used, repeatable and non-invasive method. The registration requires unchanged testing conditions (e.g., fixed hours, fixed intervals between registrations and a state of suggested relaxation). The weakness of our research is the fact that both the DC/TMD protocol and EMG do not include the functions of all of the muscles affecting jaw mobility. Sixty eight pairs of muscles above and below the mandible could be examined during head, cervical, shoulder and jaw positioning [41]. Furthermore, this and similar study designs require the researcher’s gentle touch calibration, which is necessary for clinical assessment (examination form of DC/TMD) and treatment (soft tissue mobilization). One of the main limitations of this type of study is the difficulty in establishing appropriate placebo for the control group. According to muscle physiology, a gentle touch of the skin with a hand load of about 2 g causes changes in myofascial mechanoreceptors [42]. A pressure of 5–10 g used in craniosacral therapy is clearly felt by the patient with all the consequences for the entire body [43]. In conclusion, all manual techniques applied to the face or around the face (gentle touch) are not a placebo, but rather a kind of clinical intervention. In turn, placebo modulates pain via activation of the endogenous system [44]. These effects are mirrored by psychological expectations closely connected with emotions, motivations, behavioral patterns, somatic focus and cognitions [44]. Further research on this subject is undoubtedly required.

## 5. Conclusions

Soft tissue mobilization appears to be effective in the relaxation of the masticatory muscles in patients with temporomandibular disorders-myofascial pain with referral. Despite the small group of subjects, our findings provide some evidence confirming that in patients with myofascial pain with referral, soft tissue mobilization does not improve the symmetry and synergy of the masticatory muscles limited by dental occlusion. Further studies on a larger number of samples should be conducted.

## Figures and Tables

**Table 1:** Activity (µV) of temporal, masseter, sternocleidomastoid and anterior part of the digastric muscles over 3 weeks in the entire study group (*n* = 50). The mean values, standard deviation (±SD), median (Me) and *p*-value are given. Results of the Friedman test and post hoc tests are presented.

Variables	Before 1st Treatment			After 1st Treatment			Before 2nd Treatment			After 2nd Treatment			Before 3rd Treatment			After 3rd Treatment		
	Mean	±SD	Me	Mean	±SD	Me	Mean	±SD	Me	Mean	±SD	Me	Mean	±SD	Me	Mean	±SD	Me
MT-R	108.0	48.7	99.0	82.3	27.4	77.0	84.0	28.9	81.5	79.1	33.2	70.5	86.1	32.5	83.0	77.8	27.2	76.0
MT-L	93.1	30.3	94.0	80.5	25.4	76.0	86.2	34.0	78.5	79.2	31.9	70.5	86.0	33.8	81.5	77.8	25.1	76.5
MM-R	182.7	74.3	179.5	128.9	51.7	124.0	133.0	48.1	121.0	111.2	41.8	105.0	130.0	49.9	121.0	115.6	36.7	116.5
MM-L	168.7	75.7	177.5	129.9	54.6	125.5	134.3	52.5	125.0	115.5	45.8	111.5	129.7	52.1	126.0	119.6	42.4	117.0
SCM-R	11.8	7.4	10.0	9.9	6.7	7.0	10.1	6.6	9.0	10.1	6.8	8.0	11.9	11.1	8.0	9.6	6.7	8.0
SCM-L	14.3	9.8	11.0	10.7	6.6	9.0	10.5	5.8	9.0	10.8	9.7	8.0	10.6	6.9	9.0	10.1	7.3	8.0
MD-R	20.3	8.0	19.0	16.7	7.2	15.0	18.0	9.0	16.5	15.7	7.2	14.5	17.9	7.5	17.0	15.8	5.3	15.5
MD-L	19.5	7.8	18.0	15.0	7.0	13.5	15.3	6.9	15.0	13.5	5.8	12.0	16.1	7.1	14.0	13.1	3.9	14.0
									**Post Hoc Tests/Pairwise Comparisons with the Bonferroni Correction**									
	**Friedman Test**						**Kendall’s Coefficient of Concordance**		**Before 1st and after 1st Treatment**		**Before 1st and after 2nd Treatment**		**Before 1st and after 3rd Treatment**		**Before 2nd and after 2nd Treatment**		**Before 3rd and after 3rd Treatment**	
	***Friedman Statistic (F_r_)***		***df***		***p-Value***		***W***		***p-Value***		***p-Value***		***p-Value***		***p-Value***		***p-Value***	
MT-R	25.69493		5		0.00010 *		0.10278		0.014 **		0.001 **		0.000 **		1.000		0.978	
MT-L	10.49828		5		0.06229		0.04199		0.634		0.045 **		0.154		1.000		1.000	
MM-R	75.59161		5		0.00000 *		0.30237		0.000 **		0.000 **		0.000 **		0.089		0.029 *	
MM-L	36.90299		5		0.00000 *		0.14761		0.001 **		0.000 **		0.000 **		0.426		1.000	
SCM-R	18.37309		5		0.00251 *		0.07349		0.143		0.054 **		0.001 **		1.000		0.594	
SCM-L	23.06490		5		0.00033 *		0.09226		0.242		0.002 **		0.001 **		1.000		1.000	
MD-R	22.35103		5		0.00045 *		0.08940		0.104		0.006 **		0.000 **		0.766		0.323	
MD-L	45.25414		5		0.00000 *		0.18102		0.004 **		0.000 **		0.000 **		0.167		0.132	

* *p* < 0.05 statistical significance; ** *p* < 0.05 statistical significance adjusted to the Bonferroni correction. Functional parameters: MT-R—activity of the right temporal muscle; MT-L—activity of the left temporal muscle; MM-R—activity of the right masseter muscle; MM-L—activity of the left masseter muscle; SCM-R—activity of the right sternocleidomastoid muscle; SCM-L—activity of the left sternocleidomastoid muscle; MD-R—activity of the right digastric muscle; MD-L—activity of the left digastric muscle.

**Table 2:** Activity (µV) of temporal, masseter, sternocleidomastoid and anterior part of the digastric muscles over 3 weeks in females (*n* = 37). The mean values, standard deviation (±SD), median (Me) and *p*-value are given. Results of the Friedman test and post hoc tests are presented.

Variables	Before 1st Treatment			After 1st Treatment			Before 2nd Treatment			After 2nd Treatment			Before 3rd Treatment			After 3rd Treatment		
	Mean	±SD	Me	Mean	±SD	Me	Mean	±SD	Me	Mean	±SD	Me	Mean	±SD	Me	Mean	±SD	Me
MT-R	106.2	41.5	100.0	84.2	26.5	79.0	86.7	24.4	84.0	81.8	34.5	71.0	87.0	30.8	84.0	79.4	24.4	78.0
MT-L	96.8	28.8	95.0	81.4	23.6	80.0	84.2	29.1	77.0	82.3	32.3	74.0	83.7	26.5	82.0	79.9	25.8	77.0
MM-R	189.4	79.5	188.0	126.1	46.3	122.0	132.1	42.5	121.0	114.7	44.2	105.0	134.0	46.0	127.0	120.0	32.9	120.0
MM-L	168.2	72.0	178.0	126.8	46.5	123.0	133.1	44.5	123.0	117.9	46.8	112.0	133.5	43.7	131.0	122.2	35.1	122.0
SCM-R	11.2	6.7	10.0	8.7	6.3	7.0	9.7	6.8	8.0	9.5	6.0	8.0	10.3	9.9	8.0	8.8	5.9	7.0
SCM-L	13.3	8.5	10.0	8.9	3.9	8.0	9.4	4.3	9.0	10.7	10.6	8.0	9.1	5.7	8.0	8.3	4.6	7.0
MD-R	21.1	8.3	20.0	16.7	7.5	15.0	18.7	8.8	17.0	16.1	6.2	15.0	18.3	7.4	16.0	15.8	5.2	15.0
MD-L	19.6	7.8	19.0	14.7	5.9	14.0	16.0	6.9	15.0	14.6	5.8	14.0	16.8	7.7	14.0	13.0	3.9	14.0
									**Post Hoc Tests/Pairwise Comparisons with the Bonferroni Correction**									
	**Friedman Test**						**Kendall’s Coefficient of Concordance**		**Before 1st and after 1st Treatment**		**Before 1st and after 2nd Treatment**		**Before 1st and after 3rd Treatment**		**Before 2nd and after 2nd Treatment**		**Before 3rd and after 3rd Treatment**	
	***Friedman Statistic (F_r_)***		***df***		***p-Value***		***W***		***p-Value***		***p-Value***		***p-Value***		***p-Value***		***p-Value***	
MT-R	17.87051		5		0.00311 *		0.09660		0.085		0.021 **		0.002 **		1.000		1.000	
MT-L	9.608830		5		0.08711		0.05194		0.379		0.178		0.273		1.000		1.000	
MM-R	54.16602		5		0.00000 *		0.29279		0.000 **		0.000 **		0.000 **		0.561		0.043 **	
MM-L	27.07137		5		0.00006 *		0.14633		0.003 **		0.000 **		0.004 **		0.702		1.000	
SCM-R	19.42691		5		0.00160 *		0.10501		0.021 **		0.136		0.001 **		1.000		1.000	
SCM-L	21.46962		5		0.00066 *		0.11605		0.178		0.023 **		0.000 **		1.000		1.000	
MD-R	17.80926		5		0.00320 *		0.09627		0.297		0.163		0.000 **		1.000		0.000 **	
MD-L	32.17826		5		0.00001 *		0.17394		0.006 **		0.000 **		0.000 **		0.935		0.000 **	

* *p* < 0.05 statistical significance; ** *p* < 0.05 statistical significance adjusted to the Bonferroni correction. Functional parameters: MT-R—activity of the right temporal muscle; MT-L—activity of the left temporal muscle; MM-R—activity of the right masseter muscle; MM-L—activity of the left masseter muscle; SCM-R—activity of the right sternocleidomastoid muscle; SCM-L—activity of the left sternocleidomastoid muscle; MD-R—activity of the right digastric muscle; MD-L—activity of the left digastric muscle.

**Table 3:** Activity (µV) of temporal, masseter, sternocleidomastoid and anterior part of the digastric muscles over 3 weeks in males (*n* = 13). The mean values, standard deviation (±SD), median (Me) and *p*-value are given. Results of the Friedman test and post hoc tests are presented.

Variables	Before 1st Treatment			After 1st Treatment			Before 2nd Treatment			After 2nd Treatment			Before 3rd Treatment			After 3rd Treatment		
	Mean	±SD	Me	Mean	±SD	Me	Mean	±SD	Me	Mean	±SD	Me	Mean	±SD	Me	Mean	±SD	Me
MT-R	113.2	67.0	85.0	76.8	30.2	66.0	76.3	39.2	76.0	71.3	28.8	70.0	83.5	38.3	76.0	73.5	34.8	61.0
MT-L	82.6	33.4	74.0	78.1	30.9	76.0	91.8	46.1	81.0	70.3	30.0	65.0	92.5	49.9	81.0	72.1	23.0	74.0
MM-R	163.9	55.3	172.0	136.9	66.3	126.0	135.8	63.4	132.0	101.4	33.3	105.0	118.5	60.1	115.0	103.2	44.7	106.0
MM-L	169.9	88.4	177.0	139.0	74.8	137.0	137.7	72.7	127.0	108.9	44.0	111.0	118.9	71.9	112.0	112.1	59.6	106.0
SCM-R	13.3	9.3	9.0	13.2	6.9	15.0	11.2	6.4	11.0	11.7	8.7	10.0	16.6	13.5	11.0	11.9	8.4	9.0
SCM-L	17.1	12.7	12.0	16.0	9.7	13.0	13.5	8.3	15.0	11.1	6.9	10.0	14.8	8.4	11.0	15.3	10.8	13.0
MD-R	18.2	7.2	16.0	16.8	6.7	14.0	15.9	9.7	13.0	14.5	9.9	12.0	16.9	7.9	17.0	16.0	5.8	16.0
MD-L	19.2	8.3	16.0	15.9	9.8	13.0	13.1	6.6	13.0	10.5	5.0	9.0	14.3	4.9	14.0	13.4	4.0	14.0
									**Post Hoc Tests/Pairwise Comparisons with the** ***Bonferroni Correction***									
	**Friedman Test**						**Kendall’s Coefficient of Concordance**		**Before 1st and after 1st Treatment**		**Before 1st and after 2nd Treatment**		**Before 1st and after 3rd Treatment**		**Before 2nd and after 2nd Treatment**		**Before 3rd and after 3rd soft Treatment**	
	***Friedman Statistic (F_r_)***		***df***		***p-Value***		***W***		***p-Value***		***p-Value***		***p-Value***		***p-Value***		***p-Value***	
MT-R	8.606195		5		0.12584		0.13240		0.999		0.096		0.363		1.000		1.000	
MT-L	7.989011		5		0.15685		0.12291		1.000		1.000		1.000		0.416		1.000	
MM-R	24.49115		5		0.00017 *		0.37679		0.888		0.001 **		0.001 **		0.888		1.000	
MM-L	13.35541		5		0.02027 *		0.20547		1.000		0.017 **		0.082		1.000		1.000	
SCM-R	6.531323		5		0.25790		0.10048		1.000		1.000		1.000		1.000		1.000	
SCM-L	6.008969		5		0.30535		0.09245		1.000		0.363		0.614		1.000		1.000	
MD-R	7.296380		5		0.19952		0.11225		1.000		0.153		1.000		1.000		1.000	
MD-L	20.87302		5		0.00086 *		0.32112		1.000		0.000 **		0.363		0.999		1.000	

* *p* < 0.05 statistical significance; ** *p* < 0.05 statistical significance adjusted to the Bonferroni correction. Functional parameters: MT-R—activity of the right temporal muscle; MT-L—activity of the left temporal muscle; MM-R—activity of the right masseter muscle; MM-L—activity of the left masseter muscle; SCM-R—activity of the right sternocleidomastoid muscle; SCM-L—activity of the left sternocleidomastoid muscle; MD-R—activity of the right digastric muscle; MD-L—activity of the left digastric muscle.

**Table 4:** Symmetry and synergy (%) of individual muscle groups over 3 weeks in the entire study group (*n* = 50). The mean values, standard deviation (±SD), median (Me) and *p*-value are given. Results of the Friedman test and post hoc tests are presented.

Variables	Before 1st Treatment			After 1st Treatment			Before 2nd Treatment			After 2nd Treatment			Before 3rd Treatment			After 3rd Treatment		
	Mean	±SD	Me	Mean	±SD	Me	Mean	±SD	Me	Mean	±SD	Me	Mean	±SD	Me	Mean	±SD	Me
MT ↔	81.9	12.0	83.0	83.5	12.7	87.0	83.1	11.5	85.5	85.5	9.7	84.0	82.2	11.5	84.5	86.0	11.2	89.5
MM ↔	80.1	14.4	83.0	81.3	12.5	85.0	80.5	13.0	80.5	81.3	12.1	83.5	83.4	13.5	87.0	81.1	14.2	83.5
SCM ↔	72.0	17.1	73.0	72.8	21.0	78.5	75.4	16.3	74.5	75.9	15.9	79.0	72.1	20.2	76.0	81.7	15.2	83.5
MD ↔	82.4	16.3	86.5	80.4	15.7	82.5	79.1	15.5	81.0	83.3	12.0	83.5	82.3	13.8	86.0	83.5	11.0	84.0
MT-R ↕ MM-R	60.8	17.6	59.5	59.3	17.1	58.0	59.3	17.6	62.5	61.4	19.7	61.0	59.5	17.1	58.5	63.9	19.8	68.0
MT-L ↕ MM-L	58.6	18.4	59.0	61.3	19.0	63.0	58.2	16.8	59.5	63.7	19.7	62.5	61.9	19.2	62.0	62.7	20.7	59.5
									**Post Hoc Tests/Pairwise Comparisons with the Bonferroni Correction**									
	**Friedman Test**						**Kendall’s Coefficient of Concordance**		**Before 1st and after 1st Treatment**		**Before 1st and after 2nd Treatment**		**Before 1st and after 3rd Treatment**		**Before 2nd and after 2nd Treatment**		**Before 3rd and after 3rd Treatment**	
	***Friedman Statistic (F_r_)***		***df***		***p-Value***		***W***		***p-Value***		***p-Value***		***p-Value***		***p-Value***		***p-Value***	
MT ↔	9.770749		5		0.08200		0.03908		1.000		1.000		0.456		1.000		0.122	
MM ↔	6.036088		5		0.30273		0.02414		1.000		1.000		1.000		1.000		1.000	
SCM ↔	15.84824		5		0.00729 *		0.06339		1.000		1.000		0.004 **		1.000		0.113	
MD ↔	2.116051		5		0.83286		0.00846		1.000		1.000		1.000		1.000		1.000	
MT-R ↕ MM-R	4.774697		5		0.44399		0.01910		1.000		1.000		1.000		1.000		1.000	
MT-L ↕ MM-L	6.312283		5		0.27701		0.02525		1.000		1.000		1.000		0.594		1.000	

* *p* < 0.05 statistical significance; ** *p* < 0.05 statistical significance adjusted to the Bonferroni correction. Functional parameters: MT ↔ symmetry in the activity of the temporal muscles; MM ↔ symmetry in the activity of the masseter muscles; MD ↔ symmetry in the activity of the digastric muscles; SCM ↔ symmetry in the activity of the sternocleidomastoid muscles; MTR ↕ MMR synergy of the activity of the right temporal and right masseter muscles; MTL ↕ MML synergy of the activity of the left temporal and left masseter muscles; Reference values of symmetry and synergy: <50%—unsatisfactory values; 50–70%—acceptable values; >90%—optimal values.

**Table 5:** Symmetry and synergy (%) of individual muscle groups over 3 weeks in a group of females (*n* = 37). The mean values, standard deviation (±SD), median (Me) and *p*-value are given. Results of the Friedman test and post hoc tests are presented.

Variables	Before 1st Treatment			After 1st Treatment			Before 2nd Treatment			After 2nd Treatment			Before 3rd Treatment			After 3rd Treatment		
	Mean	±SD	Me	Mean	±SD	Me	Mean	±SD	Me	Mean	±SD	Me	Mean	±SD	Me	Mean	±SD	Me
MT ↔	81.9	12.5	86.0	83.7	13.4	87.0	83.4	12.0	86.0	84.2	10.0	83.0	84.9	11.2	88.0	86.3	11.1	90.0
MM ↔	83.0	11.8	85.0	81.1	12.6	85.0	82.7	12.8	83.0	82.3	10.1	84.0	86.3	11.5	89.0	83.1	12.8	85.0
SCM ↔	72.2	18.2	75.0	74.9	22.0	81.0	75.1	16.6	74.0	75.1	15.1	78.0	75.4	19.7	81.0	81.6	16.8	84.0
MD ↔	83.0	16.2	87.0	79.2	17.5	82.0	80.2	15.5	81.0	83.8	12.5	86.0	84.8	10.8	88.0	84.2	10.3	84.0
MT-R ↕ MM-R	62.4	17.1	62.0	61.0	17.1	59.0	60.2	17.0	63.0	63.7	20.1	64.0	60.0	17.0	59.0	65.3	19.3	70.0
MT-L ↕ MM-L	59.8	18.5	59.0	63.2	19.0	65.0	60.3	16.7	62.0	65.9	16.8	65.0	60.5	17.8	57.0	64.2	20.3	60.0
									**Post Hoc Tests/Pairwise comparisons with the Bonferroni correction**									
	**Friedman Test**						**Kendall’s Coefficient of Concordance**		**Before 1st and after 1st Treatment**		**Before 1st and after 2nd Treatment**		**Before 1st and after 3rd Treatment**		**Before 2nd and after 2nd Treatment**		**Before 3rd and after 3rd Treatment**	
	***Friedman Statistic (F_r_)***		***df***		***p-Value***		***W***		***p-Value***		***p-Value***		***p-Value***		***p-Value***		***p-Value***	
MT ↔	5.164835		5		0.39610		0.02792		1.000		1.000		0.811		1.000		1.000	
MM ↔	7.729068		5		0.17182		0.04178		1.000		1.000		1.000		1.000		1.000	
SCM ↔	11.70313		5		0.03909 *		0.06326		1.000		1.000		0.028 **		1.000		1.000	
MD ↔	1.043784		5		0.95896		0.00564		1.000		1.000		1.000		1.000		1.000	
MT-R ↕ MM-R	5.261514		5		0.38481		0.02844		1.000		1.000		1.000		1.000		1.000	
MT-L ↕ MM-L	4.823668		5		0.43778		0.02607		1.000		0.811		1.000		1.000		1.000	

* *p* < 0.05 statistical significance; ** *p* < 0.05 statistical significance adjusted to the Bonferroni correction. Functional parameters: MT ↔ symmetry in the activity of the temporal muscles; MM ↔ symmetry in the activity of the masseter muscles; MD ↔ symmetry in the activity of the digastric muscles; SCM ↔ symmetry in the activity of the sternocleidomastoid muscles; MTR ↕ MMR synergy of the activity of the right temporal and right masseter muscles; MTL ↕ MML synergy of the activity of the left temporal and left masseter muscles; Reference values of symmetry and synergy: <50%–unsatisfactory values; 50–70%—acceptable values; >90%—optimal values.

**Table 6:** Symmetry and synergy (%) of individual muscle groups over 3 weeks in a group of males (*n* = 13). The mean values, standard deviation (±SD), median (Me) and *p*-value are given. Results of the Friedman test and post hoc tests are presented.

Variables	Before 1st Treatment			After 1st Treatment			Before 2nd Treatment			After 2nd Treatment			Before 3rd Treatment			After 3rd Treatment		
	Mean	±SD	Me	Mean	±SD	Me	Mean	±SD	Me	Mean	±SD	Me	Mean	±SD	Me	Mean	±SD	Me
MT ↔	82.0	10.9	82.0	83.1	10.9	82.0	82.1	10.5	82.0	89.2	8.0	93.0	74.6	8.8	78.0	84.9	11.8	87.0
MM ↔	71.9	18.1	76.0	81.9	12.6	84.0	74.2	11.8	72.0	78.5	16.7	78.0	75.2	15.6	75.0	75.5	16.9	81.0
SCM ↔	71.3	14.4	70.0	66.9	17.1	65.0	76.2	16.0	75.0	78.0	18.6	80.0	62.9	19.3	70.0	82.0	9.9	80.0
MD ↔	80.6	17.3	83.0	83.7	8.3	83.0	75.9	15.7	78.0	82.0	10.6	79.0	75.2	18.9	81.0	81.8	13.1	84.0
MT-R ↕ MM-R	56.4	19.1	53.0	54.5	16.7	53.0	56.9	19.6	62.0	54.7	17.5	58.0	58.2	18.0	58.0	59.9	21.4	58.0
MT-L ↕ MM-L	55.2	18.3	59.0	56.1	18.7	53.0	52.4	16.4	55.0	57.2	25.8	45.0	65.9	23.0	67.0	58.6	22.2	59.0
									**Post Hoc Tests/Pairwise Comparisons with the** ***Bonferroni Correction***									
	**FRIEDMAN TEST**						**Kendall’s Coefficient of Concordance**		**Before 1st and after 1st Treatment**		**Before 1st and after 2nd Treatment**		**Before 1st and after 3rd Treatment**		**Before 2nd and after 2nd Treatment**		**Before 3rd and after 3rd Treatment**	
	***Friedman Statistic (F_r_)***		***df***		***p-Value***		***W***		***p-Value***		***p-Value***		***p-Value***		***p-Value***		***p-Value***	
MT ↔	16.33630		5		0.00595 *		0.25133		1.000		1.000		1.000		1.000		0.021 **	
MM ↔	6.792035		5		0.23658		0.10449		0.316		1.000		1.000		1.000		1.000	
SCM ↔	13.10155		5		0.02245 *		0.20156		1.000		1.000		0.888		1.000		0.132	
MD ↔	6.898455		5		0.22831		0.10613		1.000		1.000		1.000		1.000		0.614	
MT-R ↕ MM-R	2.033333		5		0.84451		0.03128		1.000		1.000		1.000		1.000		1.000	
MT-L ↕ MM-L	10.07778		5		0.07306		0.15504		1.000		1.000		1.000		1.000		1.000	

* *p* < 0.05 statistical significance; ** *p* < 0.05 statistical significance adjusted to the Bonferroni correction. Functional parameters: MT ↔ symmetry in the activity of the temporal muscles; MM ↔ symmetry in the activity of the masseter muscles; MD ↔ symmetry in the activity of the digastric muscles; SCM ↔ symmetry in the activity of the sternocleidomastoid muscles; MTR ↕ MMR synergy of the activity of the right temporal and right masseter muscles; MTL ↕ MML synergy of the activity of the left temporal and left masseter muscles; Reference values of symmetry and synergy: <50%—unsatisfactory values; 50–70%—acceptable values; >90%—optimal values.
